# Mitochondrial markers (*cytochrome c oxidase subunit I* and *16S ribosomal RNA*) as supporting biomarkers for wild bird identification

**DOI:** 10.14202/vetworld.2025.1389-1399

**Published:** 2025-05-31

**Authors:** Julián Marín-Villa, Albeiro López-Herrera, Daisy A. Gómez-Ruiz, Diana C. Restrepo-Rodas, Geraldine Sánchez-Rodríguez, Cristina Úsuga-Monroy

**Affiliations:** 1 Grupo BIOGEM, Facultad de Ciencias, Universidad Nacional de Colombia sede Medellín, 050034, Medellín, Colombia; 2 Grupo BIOGEM, Facultad de Ciencias Agrarias, Universidad Nacional de Colombia sede Medellín, 050034, Medellín, Colombia; 3 Grupo GINVER, Facultad de Medicina Veterinaria, Corporación Universitaria Remington, 050010, Medellín, Colombia; 4 Grupo CYBA, Parque de la Conservación, 050024, Medellín, Colombia

**Keywords:** *16S ribosomal RNA*, *COI*, conservation genetics, mitochondrial DNA, molecular taxonomy, psittacines, wildlife forensics

## Abstract

**Background and Aim:**

Illegal wildlife trafficking is a critical threat to biodiversity, particularly in megadiverse countries such as Colombia. Birds, notably psittacines, are among the most targeted taxa. Morphological identification is often insufficient, especially when dealing with cryptic species or degraded samples. This study aimed to assess the utility of mitochondrial markers *cytochrome c oxidase subunit I* (*COI*) and *16S ribosomal RNA* (*16S rRNA*) as molecular tools for species-level identification of psittacines housed at the Conservation Park of Medellín.

**Materials and Methods:**

Six adult psittacines from the genera *Ara* and *Pionus* were selected based on availability. Blood samples were collected and genomic DNA was extracted using a commercial kit. Polymerase chain reaction amplification of partial *COI* and *16S rRNA* gene fragments was performed, followed by Sanger sequencing. Sequence identity was confirmed using BLASTn and the Barcode of Life Data System (BOLD). Phylogenetic relationships were analyzed using Neighbor-Joining, Maximum Likelihood, and Bayesian Inference approaches.

**Results:**

Molecular results showed 100% concordance with prior morphological identification for all six individuals. *COI* and *16S rRNA* sequences allowed clear species-level identification with similarity values >98%. Phylogenetic analyses for both markers yielded congruent tree topologies, with high branch support (>90%), further validating species identification. Maximum interspecific divergence for *COI* was observed between *Ara macao* and *Pionus fuscus* (0.15980), while *16S rRNA* showed lower divergence values. All generated sequences were submitted to GenBank and BOLD in accordance with findable, accessible, interoperable, reusable principles.

**Conclusion:**

This study confirms the robustness of *COI* and *16S rRNA* mitochondrial markers in accurately identifying psittacine species. The integration of molecular and morphological approaches enhances forensic investigations, facilitates biodiversity conservation, and contributes to efforts against wildlife trafficking. Expanding genetic databases for Neotropical avifauna, especially for commonly trafficked species, is imperative. Future research should adopt integrative genomic approaches involving nuclear markers to overcome the maternal inheritance limitation of mitochondrial DNA.

## INTRODUCTION

Illegal wildlife trafficking ranks among the top three most profitable global criminal enterprises, following drug and arms trafficking [1]. This illicit trade stems from various contributing factors, including social inequality, the presence of organized criminal networks, and economic disparities, and it poses a major threat to biodiversity in Colombia [2]. As a consequence, the increasing prevalence of this activity has led to severe repercussions for biodiversity and the integrity of ecosystems [3]. To combat this, environmental authorities primarily depend on morphological assessments or external phenotypic evaluations to identify wildlife. However, this method proves inadequate when dealing with cryptic species belonging to taxonomically diverse groups or when handling highly fragmented or degraded biological materials, such as skins, eggs, meat, or processed derivatives [4].

In this context, molecular diagnostic tools, particularly DNA barcoding, offer a reliable alternative for species identification. These methods rely on standardized gene regions encoded in the mitochondrial genome, including the *cytochrome c oxidase subunit I* (*COI*) and *16S ribosomal RNA* (*16S rRNA*) genes [5–7]. Mitochondrial DNA analysis plays a crucial role in the taxonomic identification of species targeted by illegal trafficking, as it enables accurate identification from minimally invasive samples such as feces, feathers, or blood. This approach is especially valuable for personnel lacking specialized expertise in morphological characterization [4, 8–10].

Colombia is recognized as one of the most biodiverse countries in the world, particularly in terms of avian species, which are notably vulnerable to the impacts of illegal wildlife trafficking [11]. Among these, psittacines are ecologically significant, contributing to ecosystem functionality through several key roles [12]. They are essential seed dispersers, aiding habitat regeneration by transporting seeds over long distances [13]. In addition, they participate in the pollination of numerous plant species by transferring pollen while feeding on flowers [14], and they assist in biological pest control by consuming insect herbivores that damage vegetation, thereby enhancing plant health [15]. These interactions – both mutualistic and antagonistic – underscore the substantial ecological influence of psittacines [16].

Despite this biodiversity, there is a clear deficiency in genetic records for psittacines in Colombia, particularly regarding the *COI* and *16S rRNA* genes. This gap significantly hampers efforts to understand and protect psittacine diversity in a country heavily affected by illegal wildlife trafficking [17].

Despite Colombia’s globally recognized avian diversity and the prevalence of psittacines in illegal wildlife trade, there remains a significant deficit in molecular data particularly mitochondrial DNA sequences for many parrot species native to the region. While morphological identification remains the standard method employed by environmental authorities, it is limited in cases involving cryptic species, degraded biological samples, or processed wildlife derivatives. Although DNA barcoding using mitochondrial markers such as *COI* and *16S rRNA* has demonstrated effectiveness in avian identification globally, its application remains underutilized and underreported in Colombian psittacine species. Moreover, the lack of comprehensive and publicly accessible genetic databases for these markers in native species constrains the utility of molecular tools in wildlife forensics, biodiversity monitoring, and conservation strategies within the country.

The present study aims to evaluate the utility of mitochondrial markers *COI* and *16S rRNA* in the molecular identification of psittacines housed at the Conservation Park of Medellín, Colombia. By combining morphological identification with molecular techniques, this study seeks to validate the accuracy and consistency of mitochondrial DNA-based taxonomy and contribute novel sequence data to global databases. Ultimately, the study endeavors to strengthen forensic and conservation applications by addressing the existing genetic information gap and supporting future initiatives to curb illegal wildlife trafficking in Colombia.

## MATERIALS AND METHODS

### Ethical approval

The Bioethics Committee for Animal Research (CIBA) of Corporación Universitaria Remington through record 08–2022 evaluated and endorsed this study.

### Study period and location

Sampling was carried out between May 2023 and June 2024 at the Conservation Park. The sampling was completed by November 2024 at La Vida Laborat-ory of the Corporación Universitaria Remington. The study was conducted at the Conservation Park, located in Medellín, Colombia, in the southeastern sector of the city, near the Olaya Herrera Airport. This conservation and environmental education center covers approximately 16 hectares in an urban setting. Medellín is situated at an altitude of 1,495 meters above sea level and has a tropical mountain climate, with an average annual temperature of 22°C.

### Selection and characteristics of individuals

For this study, six individuals from the Psittacidae family were selected. Due to ethical and logistical constraints, a convenience sampling strategy was employed. In addition, the qualitative nature of species identification rendered power analysis inapplicable. As the studied species do not exhibit obvious sexual dimorphism, sex determination was not possible. Moreover, the Conservation Park does not utilize molecular or surgical methods for sex identification. In some cases, female identification was inferred based on oviposition observation. Given the prior trafficking history of these individuals, the regional environmental authorities responsible for their relocation often lack precise information regarding their geographic origins. Each specimen was preliminarily identified based on morphological characteristics [18] and assigned a numerical ID using rings or microchips. Furthermore, all individuals had reached adulthood before their arrival at the Conservation Park; however, their exact ages remain unknown. The selected species are listed in Appendices I and II of the Convention on International Trade in Endangered Species of Wild Fauna and Flora. Although currently classified as Least Concern on the International Union for Conservation of Nature Red List of Threatened Species, the majority are reported to exhibit declining population trends (www.iucnredlist.org).

### Blood collection and DNA extraction

From each specimen, 0.5 mL of blood was collected into ethylenediaminetetraacetic acid-containing microtainer tubes through ulnar vein venipuncture. Samples were preserved and transported at refrigerated conditions (−4°C) to the La Vida Laboratory of the Corporación Universitaria Remington. Total DNA was extracted from 250 μL of peripheral blood using the HigherPurity™ DNA Extraction Kit (Canvax Biotech, Spain, Cat# AN0045-XL). The DNA was stored in 30 μL of elution buffer at −20°C until further analysis. DNA quantity and purity (260/280 and 260/230 ratios) were assessed using a Nanodrop 2,000 spectrophotometer (Thermo Fisher Scientific, USA). Samples with 260/280 ratios between 1.8 and 2.0 and 260/230 ratios above 2.0 were deemed acceptable for downstream applications. Samples that did not meet these criteria were either excluded or re-extracted.

### *COI* gene polymerase chain reaction (PCR) amplification

A partial fragment of the *COI* gene (749 bp) was amplified using two primers: BirdF1 (5’TTCTCCAACCACAAAGACATTGGCAC’3) and BirdR1 (5’ACGTGGGAGATAATTCCAAATCCTG’3) [6]. PCR reactions were carried out in 50 μL volumes containing 1 μL DNA (75 ng/μL), 0.5 μL of each primer (10 mM), 0.5 μL dNTPs (25 mM), 5 μL of 10× Taq Buffer (ExcelTaq^™^ Taq DNA Polymerase, Smobio, Taiwan, Cat# TP1000), - 0.25 μL of Taq DNA polymerase (5 U/μL), and 42.25 μL of H_2_O ultra-pure. Cycling conditions included an initial denaturation at 94°C for 5 min, followed by 40 cycles of 94°C for 30 s, 51°C for 30 s, and 72°C for 48 s, with a final extension at 72°C for 5 min. Each PCR run included a no-template control (NTC) and a positive control using known psittacine DNA to ensure amplification specificity and rule out contamination. The amplified products (725 bp) were visualized by 0.8% agarose gel electrophoresis using a Gel Doc XR+ System (Bio-Rad, USA).

### PCR amplification of *16S rRNA* gene

A 560 bp fragment of the mitochondrial *16S rRNA* gene was amplified using primers 16Sar-L (5’CGCCTGTTTATCAAAAACAT’3) and 16Sbr-H (5’CCGGTCTGAACTCAGATCACGT’3) [19]. PCR reactions were conducted in 30 μL volumes containing 1 μL DNA (75 ng/μL), 0.5 μL of each primer (10 mM), 0.5 μL dNTPs (25 mM), 5 μL of 10× Taq Buffer (ExcelTaq™ Taq DNA Polymerase, Smobio, Taiwan, Cat# TP1000), 0.25 μL Taq DNA polymerase (5 U/μL), and 22.25 μL of H_2_O ultra-pure. Cycling conditions consisted of initial denaturation at 94°C for 2 min, followed by 35 cycles at 94°C for 30 s, 53°C for 30 s, and 72°C for 48 s, with a final extension at 72°C for 1 min. Each PCR reaction included an NTC and a positive control using known psittacine DNA to verify specificity and prevent contamination. Amplified products (525 bp) were confirmed through 0.8% agarose gel electrophoresis using the Gel Doc XR+ system (Bio-Rad).

### Sequencing and data analysis

Purified PCR products were sequenced bidirectionally by Macrogen® (Seoul, Korea). Chromatograms were manually verified using FinchTV (Geospiza), and reads were trimmed at Q >20. Consensus sequences were generated using the CAP3 tool [20], with a 90% overlap identity. Ambiguities were resolved by inspecting electropherogram signal quality. Species identity based on *COI* sequences was confirmed using both the National Center for Biotechnology Information (NCBI) BLASTn tool (https://blast.ncbi.nlm.nih.gov/) and the Barcode of Life Data System (BOLD) (https://www.boldsystems.org/), while *16S rRNA* sequences were queried exclusively through BLASTn. A similarity threshold of >98% was used to confirm species-level identifi- cation. All sequences were aligned using Molecular Evolutionary Genetics Analysis (MEGA) v11 soft- ware [21] with the Multiple Unsequence Sequence Comparison by Log-Expectation algorithm, incor-porating reference sequences from GenBank and BOLD databases showing similarity values above 98%.

### Phylogenetic analysis

Cladograms based on genetic distances were constructed using the Neighbor-Joining (NJ) method with 1,000 bootstrap replicates [22]. Evolutionary distances were estimated using the Kimura 2-Parameter (K2P) model [23] in MEGA v11. In addition, phyloge- netic trees were reconstructed using the Maximum Likelihood (ML) approach with 1,000 bootstrap replicates, implemented in an open-source software package designed for phylogenetic inference using the ML method (IQ-TREE v2.3.1) [24]. The best-fit evolutionary models were selected using the ModelFinder tool in IQ-TREE by evaluating 484 nucleotide substitution models based on the Bayesian Information Criterion. The TN+I model was chosen for *COI*, and HKY+I for *16S rRNA*. Bayesian inference (BI) was performed using MrBayes v3.2.2 with the same models applied in ML analyses. Each run used four Markov chains (one cold and three heated), with sampling every 100 generations. For the *COI* gene, 500,000 generations were run until stationarity, confirmed using Tracer v1.6, with an average standard deviation of split frequencies of 0.00513. A 25% burn-in was applied before summarizing trees. For the *16S rRNA* gene, 400,000 generations were executed with an average split frequency deviation of 0.006638 and a 25% burn-in. All phylogenetic trees and cladograms were edited in iTOL v5 [25]. Complete sequence data and associated specimen metadata are available through NCBI and BOLD under accession numbers listed in Tables 1 and 2, in compliance with the Findable, Accessible, Interoperable, and Reusable data principles.

**Table 1:** Phenotypic and molecular identification of the psittacines was analyzed using the *COI* marker with respect to the molecular information deposited in the NCBI and BOLD System databases.

Specimen ID	Age	Sex	Phenotypic ID	ID NCBI	ID BOLD	NCBI match^1^		BOLD match^1^	
	
						ID species	Similarity (%)	ID species	Similarity
83310	Adult	Unidentified	*Ara ararauna*	OR537569.1	PCMED005–23	*Ara ararauna* AB570296:	100	*Ara ararauna* SIARA001–10	100
51305	Adult	Female	*Ara macao*	OR913731.1	PCMED006–23	*Ara macao* NC_045076	99.84	*Ara macao* GBMND1668–21	100
17432	Adult	Female	*Ara ararauna*	OR537570.1	PCMED004–23	*Ara ararauna* AB570296	99.84	*Ara ararauna* SIARA001–10	99.845
3987	Adult	Female	*Ara severus*	OR537573.1	PCMED001–23	*Ara severus* KF446114	100	*Ara severus* GBIR8949–19	100
7221	Adult	Unidentified	*Pionus chalcopterus*	OR537566.1	PCMED002–23	*Pionus chalcopterus* MF784450	98.94	*Pionus chalcopterus* IAvH-CT 4800	100
8475	Adult	Unidentified	*Pionus menstruus*	OR537568.1	PCMED003–23	*Pionus menstruus* JQ175860	100	*Pionus menstruus* USNMC033–10	100

*COI*=*Cytochrome c oxidase I*, ID=Identification, BOLD=Barcode of Life Data System, NCBI=National Center for Biotechnology Information. NCBI match

1 : Result of higher similarity between the problem sequence and the NCBI database. BOLD match^1^: Result of higher similarity between the problem sequence and the BOLD System database

**Table 2:** Estimates of evolutionary divergence over sequence pairs among psittacine species based on the *cytochrome c oxidase subunit I* gene.

Psittacine species	*Pionus chlacopterus*	*Ara severus*	*Pionus menstruus*	*Pionus maximiliani*	*Pionus fuscus*	*Ara militaris*	*Ara macao*	*Ara glaucogularis*	*Ara chloropterus*	*Ara ararauna*	*Ara ambiguus*
*Pionus chlacopterus*											
*Ara severus*	0.13587										
*Pionus menstruus*	0.05071	0.14108									
*Pionus maximiliani*	0.04734	0.12563	0.05926								
*Pionus fuscus*	0.04525	0.13587	0.05284	0.06667							
*Ara militaris*	0.14900	0.07109	0.15330	0.14900	**0.15980**						
*Ara macao*	0.14900	0.06229	0.15168	0.14900	**0.15980**	0.03292					
*Ara glaucogularis*	0.15167	0.07109	0.16144	0.14900	0.16254	0.06229	0.06229				
*Ara chloropterus*	0.15572	0.07332	0.15195	0.14767	0.15302	0.03904	0.04525	0.07556			
*Ara ararauna*	0.15572	0.06229	0.17536	0.15437	0.15573	0.05796	0.06229	0.06667	0.07782		
*Ara ambiguus*	0.14767	0.06778	0.14928	0.14767	0.15303	0.00844	0.02990	0.06339	0.04007	0.05689	

The analyses included 28 nucleotide sequences with a final alignment length of 539 base pairs. Evolutionary distances were conducted using the Jukes-Cantor model in MEGA v11 Data in bold represent the maximum divergence, and the underlined data represent the minimum divergence.

## RESULTS

### *COI* gene analysis

Amplification and sequencing of the *COI* gene were successfully achieved for all samples. In total, six sequences were obtained, representing five species across two genera (*Ara* and *Pionus*). The GenBank accession numbers for these sequences (NCBI IDs) are as follows: OR537569.1, OR913731.1, OR537570.1, OR537573.1, OR537566.1, and OR537568.1. Corresponding BOLD System accession numbers are as follows: PCMED005–23, PCMED006–23, PCMED004–23, PCMED001–23, PCMED002–23, and PCMED003–23.

Sequence alignment was performed using 28 sequences with a final aligned length of 539 base pairs, following quality control verification in FinchTV. This dataset included the six novel sequences generated in the present study along with reference sequences obtained from the NCBI and BOLD databases. The average nucleotide composition was as follows: Adenine (A) 25.8%, guanine (G) 16.2%, thymine (T) 25.8%, and cytosine (C) 32.2%. The molecular identifications obtained were in complete agreement (100%) with prior morphological identifications (Table 1).

**Figure 1:**
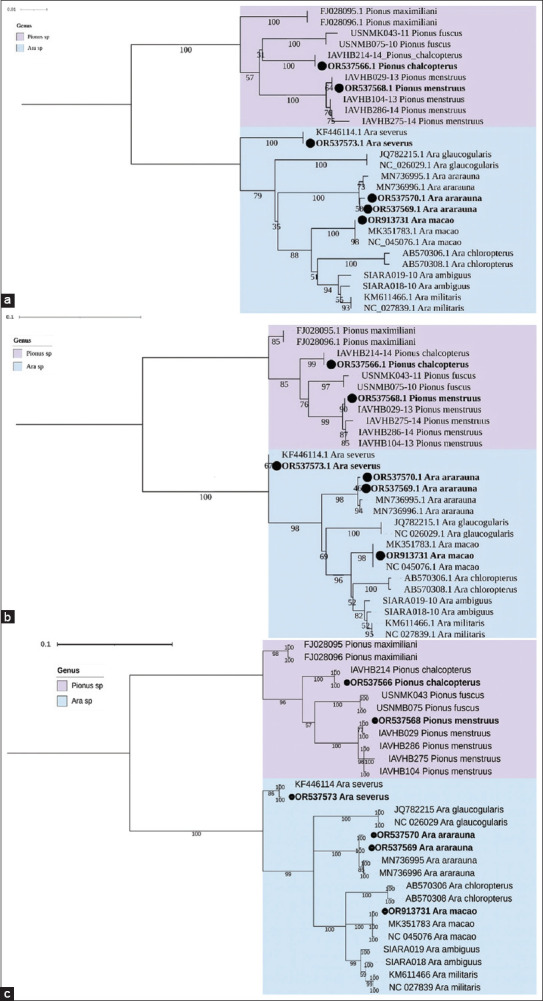
Cladogram and phylogenetic trees of the *Ara* and *Pionus* genera based on partial mitochondrial *COI* gene sequences (539 bp). (a) Neighbor-Joining cladogram constructed with 1,000 bootstrap replicates using the Kimura 2-Parameter model. (b) Maximum Likelihood phylogenetic tree with 1,000 bootstrap replicates under the TN+I substitution model. (c) Bayesian inference tree generated over 500,000 generations using the TN+I model. Black dots (•) denote the sequences corresponding to psittacines sampled from the Conservation Park of Medellín. A total of 22 reference sequences from the NCBI and BOLD System databases were included in the analysis. NCBI=National center for biotechnology information, BOLD=Barcode of Life Data System.

Similarly, analysis of the cladogram (Figure 1a) revealed that all branches containing the individuals sequenced in this study exhibited 100% support values. In the phylogenetic tree generated using the ML method (Figure 1b), branch support values exceeded 90%, indicating high confidence in the inferred relationships. Moreover, the tree constructed through BI (Figure 1c) was congruent with those obtained using the NJ and ML methods and demonstrated 100% branch support across all relevant nodes.

The highest interspecific divergence observed for the *COI* gene was 0.15980, recorded between *Ara macao* and *Pionus fuscus*, as well as between *Ara militaris* and *P. fuscus*. In contrast, the lowest divergence was noted between *Ara chloropterus* and *A. militaris*, with a value of 0.03904 (Table 2). These findings are consistent with the phylogenetic relationships illustrated in Figure 1. Regarding intraspecific variation, the greatest divergence was observed within *Ara ambiguus* (0.0094) and *Pionus menstruus* (0.0045).

### *16S rRNA* gene analysis

All samples evaluated for the *16S* *rRNA* gene showed successful amplification and sequencing. A total of six sequences were obtained, corresponding to five species across two genera (*Ara* and *Pionus*). The GenBank accession numbers for these sequences (NCBI IDs) are as follows: OR819873.1, OR819872.1, OR819871.1, OR819874.1, OR819875.1, and OR819876.1. As no corresponding data for the *16S rRNA* gene are available in the BOLD System, these sequences were not deposited there.

Sequence alignment included 22 sequences with a final aligned length of 531 base pairs, after quality verification in FinchTV. This dataset comprised the six newly generated sequences along with reference sequences retrieved from NCBI. The average nucleotide composition was as follows: Adenine (A) 30.8%, guanine (G) 20.9%, thymine (T) 19.7%, and cytosine (C) 28.6%. As with the *COI* gene, molecular identification based on the *16S rRNA* marker was in complete agreement (100%) with prior morphological identifications (Table 3).

**Table 3:** Phenotypic and molecular identification of psittacine individuals based on the *16S rRNA* marker, using reference sequences deposited in the GenBank database. Due to the absence of *16S rRNA* data in the BOLD System, comparisons were conducted exclusively using GenBank.

Specimen ID	ID NCBI	Phenotypic ID	NCBI match^1^	

			ID species	Similarity (%)
83310	OR819873	*Ara ararauna*	*Ara ararauna* KF946546.1	98.98
51305	OR819872	*Ara macao*	*Ara macao* NC_045076.1	100
17432	OR819871	*Ara ararauna*	*Ara ararauna* EU197110.1	100
3987	OR819874	*Ara severus*	*Ara severus* KF946546.1	98.98
7221	OR819875	*Pionus chalcopterus*	*Pionus chalcopterus* MF784450.1	99.81
8475	OR819876	*Pionus menstruus*	*Pionus menstruus* KX925978.1	99.50

ID=Identifier, BOLD=Barcode of Life Data System, NCBI=National Center for Biotechnology Information, *16S rRNA*=*16S ribosomal RNA*. NCBI match

1 : Result of higher similarity between the problem sequence and the NCBI database

**Figure 2:**
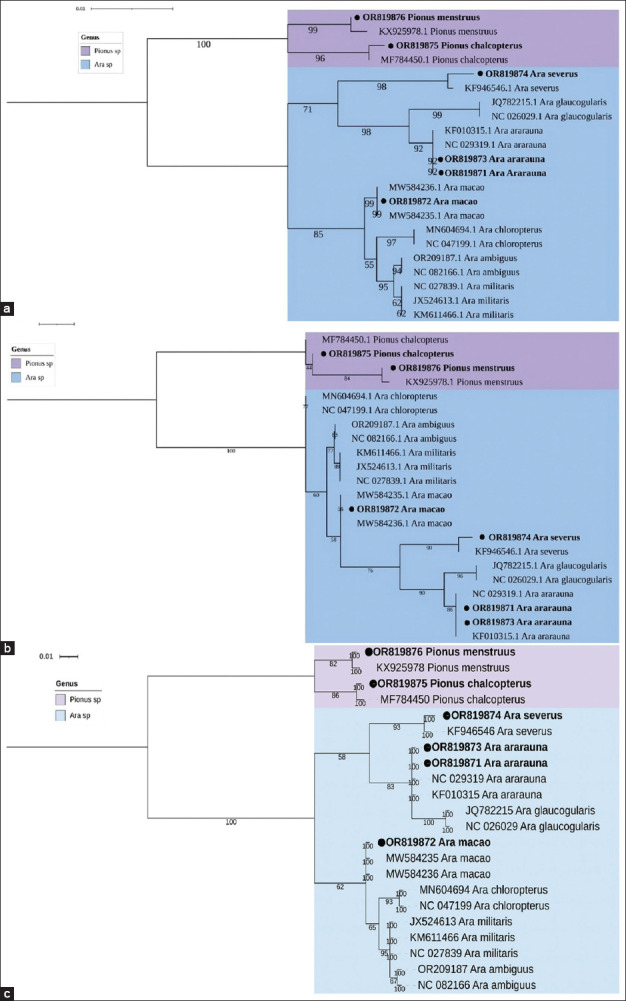
Cladogram and phylogenetic trees of the *Ara* and *Pionus* genera based on partial mitochondrial *16S ribosomal RNA* gene sequences (531 bp). (a) Neighbor-Joining cladogram constructed with 1,000 bootstrap replicates using the Kimura 2-Parameter model. (b) Maximum Likelihood phylogenetic tree generated with 1,000 bootstrap replicates using the HKY+I substitution model. (c) Bayesian inference tree constructed over 400,000 generations using the TN+I model. Black dots (•) represent the sequences of psittacines sampled from the Conservation Park of Medellín. A total of 16 reference sequences from the NCBI database were included in the analysis. NCBI=National center for biotechnology information.

In the cladogram analysis (Figure 2a), the minimum branch support value observed among the studied individuals was 92% for *Ara ararauna*, while maximum support values of 99% were recorded for *Ara. macao* and *P. menstruus*. In the phylogenetic tree constructed using the ML method (Figure 2b), the lowest branch support value was 84%. All individuals clustered with their respective conspecifics based on reference sequences from NCBI. The Bayesian phylogenetic tree (Figure 2c) demonstrated uniformly high branch support values (100%) and exhibited a topology congruent with those obtained from the NJ and ML methods.

Based on the *16S rRNA* gene analysis, the highest interspecific divergence was observed between *Ara glaucogularis* and *Pionus chalcopterus* (0.06509), while the lowest divergence was recorded between *A. ambiguus* and *A. militaris* (0.00189) (Table 4). In addition, the highest intraspecific divergence was found within the *Ara severus* group, with a value of 0.0038.

**Table 4:** Estimates of evolutionary divergence over sequence pairs among psittacine species based on the *16S rRNA* gene.

Psittacine species	*Pionus chlacopterus*	*Pionus menstruus*	*Ara severus*	*Ara chloropterus*	*Ara militaris*	*Ara ambiguus*	*Ara macao*	*Ara glaucogularis*	*Ara ararauna*
*Pionus chlacopterus*									
*Pionus menstruus*	0.01883								
*Ara severus*	0.05377	0.05377							
*Ara chloropterus*	0.04811	0.04811	0.03774						
*Ara militaris*	0.05566	0.05189	0.03396	0.00755					
*Ara ambiguus*	0.05566	0.05189	0.03396	0.00755	0.00189				
*Ara macao*	0.05377	0.05000	0.02830	0.00943	0.00566	0.00566			
*Ara glaucogularis*	**0.06509**	0.06415	0.03019	0.03962	0.03396	0.03396	0.03019		
*Ara ararauna*	0.06132	0.06132	0.02642	0.03208	0.02830	0.02830	0.02642	0.01132	

*16S rRNA*=*16S ribosomal RNA.* The analysis included 22 nucleotide sequences with a final alignment length of 531 base pairs. Evolutionary distances were calculated using the Jukes-Cantor model in MEGA v. 11. Bolded values indicate the highest observed divergence, while underlined values denote the lowest.

## DISCUSSION

The *COI* and *16S rRNA* gene sequences demonstrated strong discriminatory capacity in this study and proved effective for psittacine species identification, both in terms of sequence similarity with reference databases and phylogenetic resolution through NJ cladograms. However, two key criteria are essential to evaluate the effectiveness of such molecular techniques. First, sufficient sequence divergence must exist to distinguish closely related species. Second, reference databases must comprehensively represent the taxonomic group in question [26]. Both conditions were satisfactorily met in the present study.

Mitochondrial gene sequencing has previously been employed to identify birds involved in illegal wildlife trafficking, their derivatives, and even individuals implicated in aviation-related incidents [4, 6, 26–30]. Molecular approaches are thus considered valuable tools in circumstances where morphological identification is difficult, highlighting their applicability in forensic and ecological contexts [31]. In our study, samples from captive psittacines confiscated due to trafficking and held at a Colombian conservation park were successfully classified using mitochondrial markers (*COI* and *16S rRNA*).

Previous studies using these markers for psittacine identification have reported similar success rates. For example, Gonçalves *et al*. [9] identified 57 of 58 individuals correctly, though one specimen presented conflicting results: The BOLD System identified it as *Megascops choliba*, while GenBank classified it as *Bubo virginianus*. This inconsistency likely resulted from the absence of *M. choliba* *16S rRNA* sequences in GenBank, emphasizing the importance of comprehensive and well-curated sequence databases to avoid misidentification.

Mitochondrial DNA analysis remains a robust method for species identification [32], as corroborated by our findings. Using two mitochondrial genes and three phylogenetic methods, we accurately identified psittacines from the genera *Ara* and *Pionus*. All phylogenetic trees showed congruent topologies and high branch support values (>80%) (Figures 1 and 2). However, exclusive reliance on mitochondrial DNA presents limitations, given its maternal inheritance. Phenomena such as hybridization and incomplete lineage sorting may obscure species-level resolution. Consequently, several authors advocate for an integrative taxonomy that combines mitochondrial and nuclear genetic markers to enhance identification accuracy [33].

While the *16S rRNA* gene has historically been used for genus-level classification, advancements in analytical methods and database coverage now enable its application for species-level resolution [34]. Although the *16S rRNA* gene displayed lower interspecific divergence than *COI* in this study, it still provided accurate identification, as previously demonstrated by Gao *et al*. [35]. The *16S rRNA* amplicon yielded 49 parsimony-informative sites (out of 531 bp), compared with 123 sites for *COI* (out of 539 bp). Despite this, both markers consistently matched the birds’ phenotypic identifications. This validates the comprehensive strategy employed, incorporating both morphological and molecular analyses for future identification of trafficked psittacines, which are among the most frequently targeted species.

Notwithstanding the maternal inheritance limitations of mitochondrial DNA, both *COI* and *16S rRNA* were effective in species discrimination in this study. Nonetheless, the importance of combining mitochondrial with nuclear markers is underscored to mitigate the risk of misclassification due to hybridization or incomplete lineage sorting [36, 37]. A holistic approach, leveraging all available genetic data, provides a more accurate and robust framework for taxonomic resolution.

It has been estimated that one-third of international wildlife trafficking cases fail to reach a legal conclusion due to the absence of definitive species identification [38]. Thus, integrating molecular tools can significantly enhance forensic investigations and contribute to the prosecution of wildlife crimes. Moreover, assigning genetic origins could help trace trafficking routes [39], although such applications demand more detailed population-level analyses. Continued research is warranted in this area. Captive animal populations, such as those in conservation parks and rehabilitation centers, represent a critical resource for establishing genetic baselines to support *in situ* and *ex situ* conservation strategies [40].

While the sample size in this study was limited, it provided a preliminary validation of mitochondrial markers for psittacine identification. However, the small number of individuals may not capture the full genetic diversity of captive populations, potentially introducing bias. Future studies should employ stratified or randomized sampling designs to improve representativeness and reduce selection bias. Although power analysis was not applicable due to the qualitative nature of species identification, subsequent research should aim for larger sample sizes to enable a more accurate evaluation of intraspecific variability. In this context, zoological institutions, wildlife evaluation centers, and conservation facilities in Colombia and beyond play an essential role by providing access to biological material and facilitating ongoing scientific investigations.

## CONCLUSION

This study demonstrated the efficacy of mitochondrial markers *COI* and *16S rRNA* as reliable molecular tools for the accurate identification of psittacines housed at the Conservation Park of Medellín, Colombia. Both markers yielded 100% concordance with phenotypic identifications and facilitated species-level classification across two genera (*Ara* and *Pionus*). The phylogenetic trees constructed using NJ, ML, and BI methods exhibited congruent topologies with high branch support values (>90%), further validating the robustness of the approach.

A key strength of this study lies in its integration of morphological and molecular data, which enhances taxonomic accuracy and mitigates the limitations of single-method identifications. In addition, the successful sequencing and alignment of all six samples, combined with their high similarity to reference sequences from GenBank and BOLD (for *COI*), underscore the diagnostic power of mitochondrial DNA for wildlife forensics and conservation biology.

However, the study was limited by its small sample size and the absence of nuclear genetic data. While the qualitative nature of species identification justified the use of a convenience sampling strategy, the limited number of individuals may not fully capture the genetic variability of trafficked psittacines. Furthermore, reliance on maternally inherited mitochondrial markers, although effective, may overlook hybridization events or incomplete lineage sorting, which can confound species-level resolution.

To address these limitations, future studies should incorporate larger, geographically stratified samples and include nuclear markers to complement mitochondrial data. Expanding the representation of native Neotropical bird species in genetic databases will also improve identification accuracy. Moreover, integrating molecular tools into wildlife law enforcement protocols can support forensic investigations, trace trafficking routes, and aid in biodiversity conservation efforts.

This study provides foundational evidence supporting the use of *COI* and *16S rRNA* genes for molecular identification of psittacines in Colombia. It highlights the importance of genetic tools in combating illegal wildlife trafficking and offers a replicable model for applying molecular taxonomy in conservation and forensic science contexts.

## Data availability

All data analyzed during the current study are available from the corresponding author on request.

## AUTHORS’ CONTRIBUTIONS

JM: Processing of samples, molecular analyses, information analysis, and drafted and reviewed the manuscript. AL: Conceptualization, data analysis, and reviewed and edited the manuscript. DAG: Data analysis and drafted, reviewed, and edited the manuscript. DCR: Conceptualization, morphological identification, and drafted and reviewed the manuscript. GS: Monitoring, capture, processing of samples, and drafted and reviewed the manuscript. CU: Molecular review, project administration, and drafted, edited, and reviewed the manuscript. All authors have read and approved the final manuscript.
